# Classification of imbalanced oral cancer image data from high-risk population

**DOI:** 10.1117/1.JBO.26.10.105001

**Published:** 2021-10-23

**Authors:** Bofan Song, Shaobai Li, Sumsum Sunny, Keerthi Gurushanth, Pramila Mendonca, Nirza Mukhia, Sanjana Patrick, Shubha Gurudath, Subhashini Raghavan, Imchen Tsusennaro, Shirley T. Leivon, Trupti Kolur, Vivek Shetty, Vidya Bushan, Rohan Ramesh, Tyler Peterson, Vijay Pillai, Petra Wilder-Smith, Alben Sigamani, Amritha Suresh, Moni Abraham Kuriakose, Praveen Birur, Rongguang Liang

**Affiliations:** a The University of Arizona, Wyant College of Optical Sciences, Tucson, Arizona, United States; b Mazumdar Shaw Medical Centre, Bangalore, India; c KLE Society Institute of Dental Sciences, Bangalore, India; d Mazumdar Shaw Medical Foundation, Bangalore, India; e Biocon Foundation, Bangalore, India; f Christian Institute of Health Sciences and Research, Dimapur, India; g University of California Beckman Laser Institute and Medical Clinic, Irvine, California, United States; h Cochin Cancer Research Center, Kochi, India

**Keywords:** oral cancer, mobile screening device, imbalanced multi-class datasets, deep learning, ensemble learning

## Abstract

**Significance:** Early detection of oral cancer is vital for high-risk patients, and machine learning-based automatic classification is ideal for disease screening. However, current datasets collected from high-risk populations are unbalanced and often have detrimental effects on the performance of classification.

**Aim:** To reduce the class bias caused by data imbalance.

**Approach:** We collected 3851 polarized white light cheek mucosa images using our customized oral cancer screening device. We use weight balancing, data augmentation, undersampling, focal loss, and ensemble methods to improve the neural network performance of oral cancer image classification with the imbalanced multi-class datasets captured from high-risk populations during oral cancer screening in low-resource settings.

**Results:** By applying both data-level and algorithm-level approaches to the deep learning training process, the performance of the minority classes, which were difficult to distinguish at the beginning, has been improved. The accuracy of “premalignancy” class is also increased, which is ideal for screening applications.

**Conclusions:** Experimental results show that the class bias induced by imbalanced oral cancer image datasets could be reduced using both data- and algorithm-level methods. Our study may provide an important basis for helping understand the influence of unbalanced datasets on oral cancer deep learning classifiers and how to mitigate.

## 1 Introduction

Oral cancer is a common disease, particularly in low- and middle-income countries. Early detection of oral cancer is believed to be the most effective way to prevent it. Automatic oral cancer image classification algorithms based on machine learning enable a system to learn from previous data and, based on the learning, predict and give results on new unseen data. However, oral cancer datasets captured from high-risk populations are often unbalanced since there are many more normal cases than benign, premalignant, and malignant cases. Due to the influence of the larger majority classes featured on machine learning training framework, the classifier results may be biased toward the over-represented class (or classes). The discrimination of minority classes is also of great clinical significance. For example, a false positive result for benign lesions in cancer screening will result in unnecessary psychological stress, medical procedures to patients, and increased clinical workloads. Similarly, classifiers also need high sensitivity because they are designed for cancer screening. Therefore, one of the big challenges is handling class imbalance, especially for multiple category classification.

Convolutional neural networks have shown promising performance in the field of biomedical imaging. Its architecture contains two or more convolutional layers to map input data to new representations or make predictions, which is also affected by this class imbalance issue. Previous research^1^ shows the net gradient that is responsible for updating the model’s weight is dominated by the majority class (or classes). This increases the error of the minority class (or classes) in class imbalanced scenarios. Deep learning methods for addressing class imbalance can be categorized into data level and algorithm level.

Data-level approaches attempt to reduce the level of imbalance through data sampling methods. Xie et al.^2^ overcame the influence from the imbalanced histopathological images by turning images up and down, right and left, and rotating for deep learning-based breast cancer analysis. Ismael et al.^3^ used data augmentation to solve the problem of class imbalance for deep learning-based brain cancer magnetic resonance imaging image classification. Han et al.^4^ augmented the training examples based on the ratios of imbalanced classes to solve the imbalanced class problem for deep learning-based breast cancer histopathological image classification. Undersampling methods are also used to solve the imbalanced cancer image classification problem. Sui et al.^5^ trained a support vector machine lung nodule classifier based on a combination of undersampling and oversampling. Zhang et al.^6^ used a cluster-based undersampling method to overcome the imbalance problem in breast cancer classification.

Algorithm-level methods were also developed for imbalanced deep learning and commonly implemented with a class weight or penalty for handling class imbalance in order to reduce bias toward the majority group. Lin et al.^7^ presented a new loss function named focal loss, which reshapes the cross-entropy (CE) loss to reduce the impact that easily classified samples have on the loss. This new loss function not only reduces the class imbalance problem but also samples difficult to classify. Zhou et al.^8^ used the focal loss function to train the deep learning model for optical diagnosis of colorectal cancer. Tran et al.^9^ used both focal loss and data augmentation for imbalanced lung nodule classification. Cost-sensitive learning, reweighting of training data to assign larger weights to minority classes, is also widely used in deep learning to solve the class imbalance problem.^10^^–^^12^ Additionally, ensemble methods, which combine multiple classifiers to achieve better performance than any of the single classifiers, have been proposed to address the imbalanced medical image analysis problem in the deep learning framework.^13^^–^^16^

In this paper, we use both data- and algorithm-level methods to improve the neural network performance of oral cancer image classification with imbalanced multi-class datasets captured from high-risk populations during oral cancer screening programs using our customized devices.^17^^,^^18^ The challenge of data imbalance is common in oral-cancer image classification, researchers have acknowledged this problem and attempted to solve it in their previous studies.^19^^,^^20^ We have investigated and compared the performance of different approaches for imbalanced oral cancer image classification. Our experimental results show that class bias could be reduced by combining multiple data- and algorithm-level methods, and the performance metrics adopted in the study could be used to evaluate the imbalance issue.

The class bias discussed above is an uneven representation of classes in the training data. It may lead to bias toward the over-represented class. Classifier bias is different from class bias, and it is the difference between the predicted value of the model and the expected correct value. The variance error is the variability of a model prediction for a given data point. There is a trade-off between bias and variance of a classifier, high variance error leads to overfitting, and high bias error leads to underfitting of the model.

## 2 Methods

### 2.1 Imbalanced Oral Cheek Mucosa Image Dataset

**Fig. 1:**
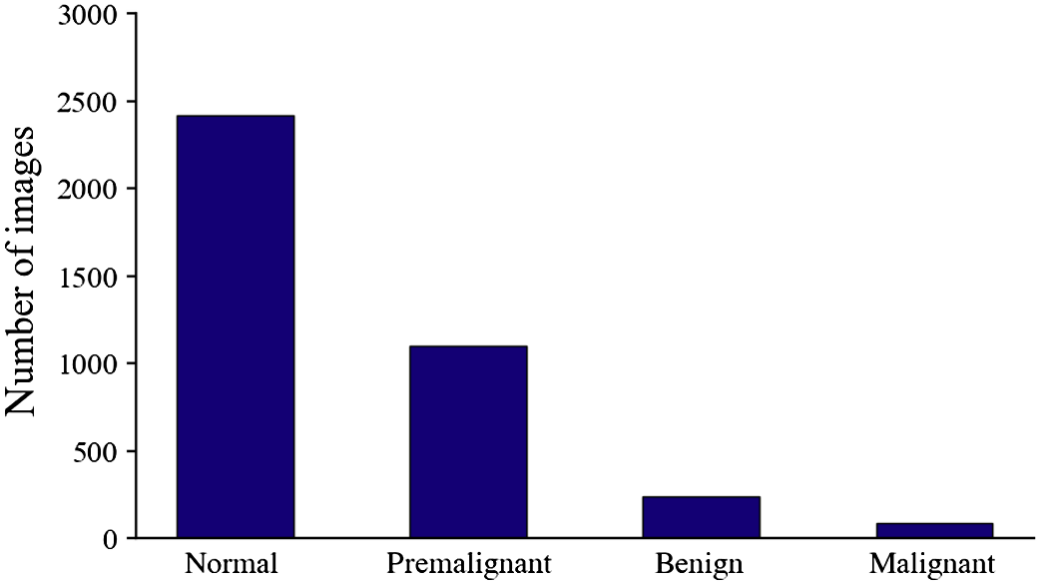
Distribution of imbalanced oral cheek mucosa image dataset collected from high-risk population.

The oral cancer image dataset was captured among patients attending the outpatient clinics of Department of Oral Medicine and Radiology at KLE Society Institute of Dental Sciences, Head and Neck Oncology Department of Mazumdar Shaw Medical Center, and Christian Institute of Health Sciences and Research, India. We used our customized mobile oral screening device to collect the oral cancer cheek mucosa image dataset.^17^^,^^18^ The dataset collected from this high-risk population is imbalanced, as it contains 3851 total polarized white light cheek mucosa images, 2417 of which are normal, 1100 are premalignant cases, 243 are benign cases, and 91 are malignant cases (see Fig. 1).

### 2.2 Network Training

We used a VGG19^21^ model pretrained with ImageNet in all experiments for fair comparison. The experiments were implemented in python platform using Tensorflow and Keras tool. For each experiment, the dataset was randomly split into training and validation to perform a fourfold cross validation, and results from the folds were averaged. The batch size was 32, learning rate was 0.001, the epoch number was 300, and Adam optimizer was used for each experiment.

### 2.3 Data-Level Approach

Data-level approaches modify the datasets to balance distributions. The two most common techniques are oversampling and undersampling. These methods decrease the level of imbalance by modifying the training distributions; for example, random oversampling duplicates samples from the minority group and random undersampling (RUS) discards random samples from majority group. Some studies indicate oversampling may increase the probability of overfitting, and undersampling may cause underfitting.^22^^–^^24^ Data augmentation is a commonly used oversampling method to amplify the minority classes by turning images up and down, left and right, and applying a random rotation. A more effective solution is combining both oversampling and undersampling techniques.

### 2.4 Algorithm-Level Approach

Algorithm-level approaches modify learning algorithms to alleviate, which includes weight balancing, new loss functions, and ensemble learning.

Weight balancing balances the dataset by adjusting the weight that each training class carries when computing the loss during training. Usually, each class carries equal 1.0 weight in the loss function. But we might want minority classes to hold more weight, and the weighting factor will be set by inverse class frequency. Weighted cross entropy loss is defined as

$$L_{\mathrm{wce}}=-\alpha_{t}\;\log(p_{t}),$$

α is the weighting factor and $p_{t}$ represents the model’s estimated probability.

Focal loss is first introduced to address the object detection scenario, in which there is an extreme imbalance between foreground and background classes and is used later on to lessen imbalance during classification. By adding a modulating factor ${\left(1-p_{t}\right)}^{\gamma }$ to the conventional cross entropy loss, with tunable focusing parameter γ, and weighting factor α, the focal loss is defined as

$$\mathrm{FL}(p_{t})=-\alpha_{t}{\left(1-p_{t}\right)}^{\gamma }\;\log(p_{t}).$$

Focal loss weighs the contribution of each instance to the loss based on the classification error. The contribution to the loss decreases if the instance is already classified correctly by the deep learning model. It also weighs the contribution of each class to the loss in a more balanced way.

**Fig. 2:**
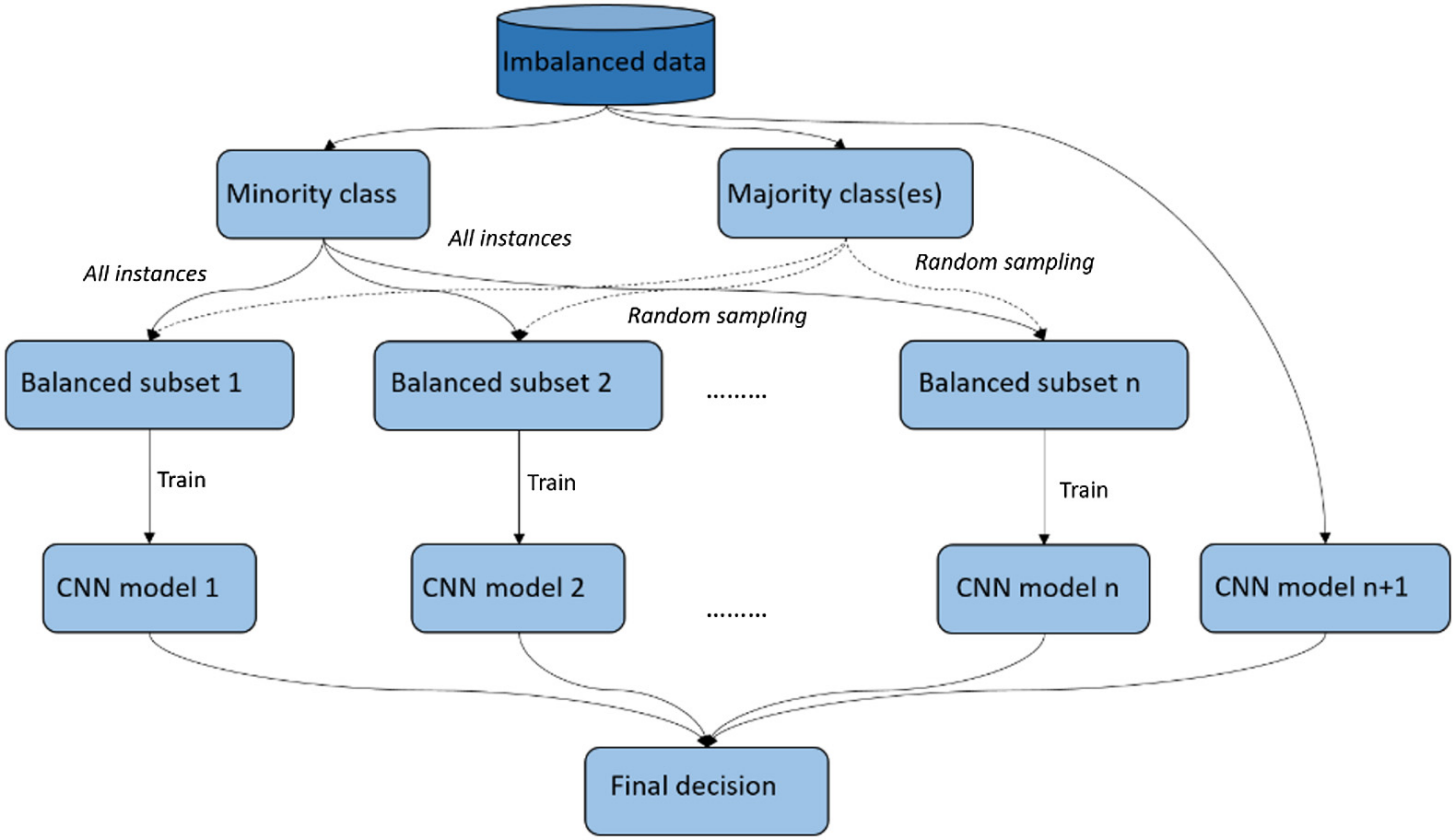
The bootstrap aggregation ensemble method for imbalanced dataset.

In addition to a single classifier, an ensemble of classifiers can also be used to reduce the class imbalance problem. In this study, we used the bootstrap aggregation ensemble method^25^ to create multiple balanced subdatasets by repeatedly resampling majority cases and combining selected majority cases with minority cases and then used the balanced subdatasets to train multiple classifiers. The results from multiple nets were combined for a final decision (see Fig. 2). Ensemble methods are usually computationally expensive, which requires more time, memory, and computing resources.

### 2.5 Performance Metrics

The most frequently used metrics to evaluate classification results are accuracy and error rate. Both are insufficient when working with imbalanced class datasets since the results are dominated by the majority classes and fail to reflect the performance of minority classes. We used confusion metrics, precision, recall, receiver operating characteristics (ROC) curve, and area under the ROC curve, which can better evaluate imbalanced data problem.

ROC curve plots true positive rate over false positive rate. Since this is a multi-class dataset, the ROC curve for each class is calculated as one class versus all other classes. The microaveraging and macroaveraging methods are used to combine the multi-class ROC. Macroaveraging averages the performances of each individual class, and microaveraging considers each element of the label indicator matrix as a binary prediction. As an example, the macroaveraging k-classes precision is calculated by

$${\text{precision}}_{\text{macro}}=\frac{{\mathrm{pr}}_{1}+{\mathrm{pr}}_{2}+\ldots +{\mathrm{pr}}_{k}}{k}.$$

And the microaveraging k-classes precision is calculated as

$${\text{precision}}_{\text{micro}}=\frac{{\mathrm{TP}}_{1}+{\mathrm{TP}}_{2}+\ldots +{\mathrm{TP}}_{k}}{\left({\mathrm{TP}}_{1}+{\mathrm{TP}}_{2}+\ldots +{\mathrm{TP}}_{k})+({\mathrm{FP}}_{1}+{\mathrm{FP}}_{2}+\ldots +{\mathrm{FP}}_{k}\right)}.$$

## 3 Results

We did experiments to evaluate the performance of the aforementioned methods with our imbalanced oral cheek mucosa image dataset.

We first trained the original dataset with a pretrained VGG19 net and conventional CE loss to get a baseline result. The result shows that (second column Table 1) although the overall accuracy is 81%, the result is dominated by majority class. The classifier did not perform well on minority classes, especially the benign cases; the most of the benign cases were incorrectly classified. It also shows that the performance of deep learning-based oral cancer image classification is influenced by imbalanced datasets. The minority class “cancer/malignancy” has much higher accuracy than “benign” maybe because malignant lesions have clear features and are easier to classify, whereas features of benign lesions appear similar to normal and suspicious cases, which adds extra difficulty on top of an insufficient quantity of data.

**Table 1:** The results table of experiments in the study.

Dataset/loss function	Original/CE	Original/weight-balanced CE	Original/focal loss	Oversampled/CE	RUS and oversampled/CE
F1-score (benign)	0.11	0.17	0.30	0.62	0.68
F1-score (malignancy)	0.80	0.80	0.80	0.81	0.84
F1-score (normal)	0.89	0.88	0.89	0.94	0.95
F1-score (premalignancy)	0.74	0.72	0.72	0.71	0.73
Recall (benign)	0.10	0.12	0.23	0.57	0.69
Recall (malignancy)	0.75	0.67	0.67	0.71	0.75
Recall (normal)	0.94	0.92	0.93	0.92	0.92
Recall (premalignancy)	0.73	0.74	0.71	0.82	0.80
Precision (benign)	0.36	0.35	0.41	0.67	0.69
Precision (malignancy)	0.86	0.98	0.98	0.94	0.95
Precision (normal)	0.85	0.85	0.86	0.95	0.98
Precision (premalignancy)	0.75	0.71	0.73	0.63	0.67
Macroaverage precision	0.70	0.73	0.75	0.80	0.83
Macroaverage recall	0.62	0.61	0.64	0.76	0.79
Macroaverage F1-score	0.64	0.65	0.68	0.77	0.81
Total accuracy	0.81	0.80	0.81	0.78	0.81
Balanced accuracy	0.62	0.61	0.64	0.75	0.80

Before applying the data-level approach, we tried algorithm-level methods (weight balancing and focal loss) on the original imbalanced dataset. The class weight assigned to each category for weight balancing is inversely proportional to class frequencies. The focusing parameter and weighting factor of focal loss used this time were 2 and 0.25, respectively, as recommended by the paper.^7^ The settings such as split ratio, batch size, and learning rate were the same as before for a fair comparison. The results of applying the weight balancing and focal loss algorithms to the original imbalanced dataset are shown in the third and fourth columns of Table 1. From the results, we can see that the performance of classifiers on the minority hard-to-classify category benign was slightly improved by algorithm-level approaches but most of the benign cases were still misclassified. The results also indicate that it is insufficient to solve the highly imbalanced oral image dataset with algorithm-level approaches only.

**Fig. 3:**
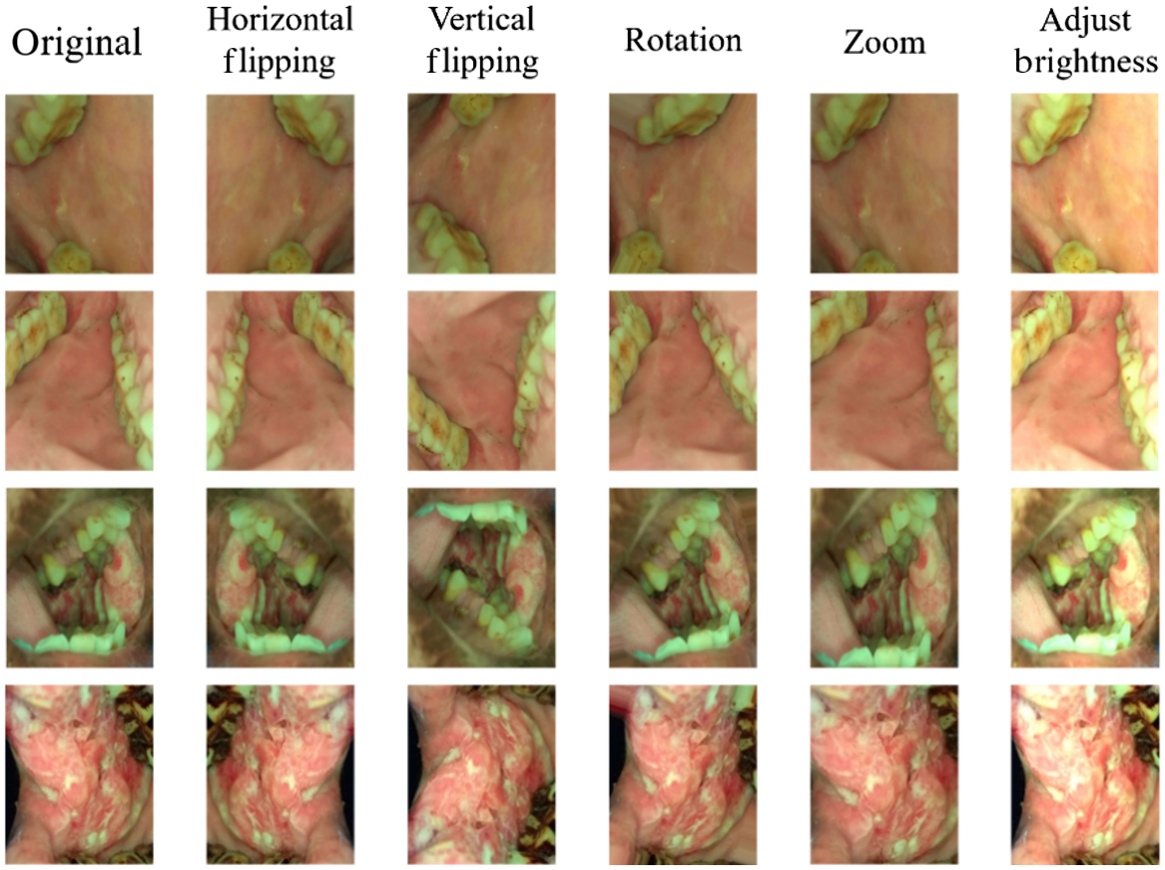
Data augmentation examples of our oral cheek mucosa dataset.

Data augmentation is widely used in object detection, segmentation, and image classification to increase the amount of input data by random perturbation. Common image data augmentations include padding, horizontal and vertical flipping, random cropping, and rotating. Usually, data augmentation will act on all the training data to increase training set diversity to make the model adapt to a variety of conditions. We augmented the training examples based on the ratios of imbalanced classes to oversample the dataset. The examples of data augmentation are shown in Fig. 3.

We used CE loss to train the oversampled dataset, using the same settings as before for a fair comparison. Applying data augmentation resulted in improved classifier performance for minority classes, as shown in the fifth column of Table 1.

Then we tried to combine both oversampling and undersampling for our application. First, we randomly undersampled the majority classes, “normal” and “premalignancy,” to 600. Then we oversampled the minority classes, benign and malignancy, using data augmentation to generate a balanced dataset. The balanced dataset was trained with CE loss for comparison. The result of combining both oversampling and undersampling is shown in sixth column Table 1. We can see the performance of the classifier on the minority classes was better then use data augmentation alone.

**Fig. 4:**
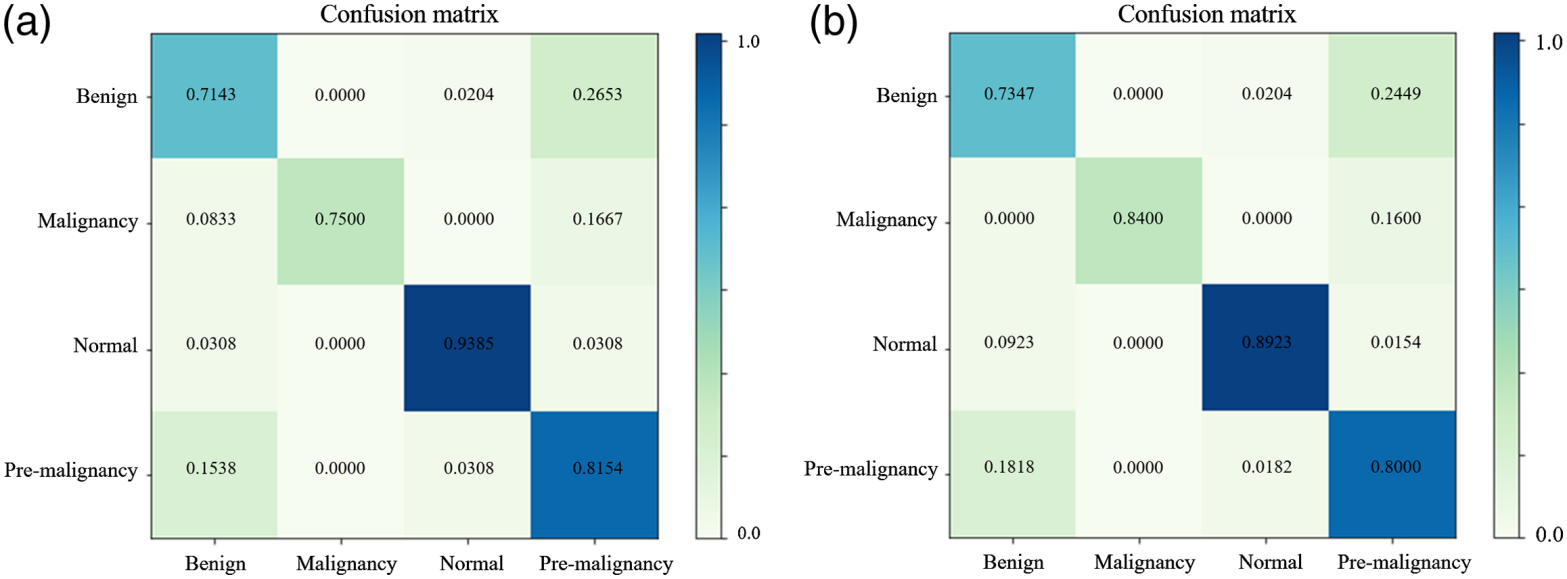
Confusion matrix: (a) trained with RUS and data augmented balanced dataset using focal loss and (b) trained with bootstrap aggregation ensemble method.

**Fig. 5:**
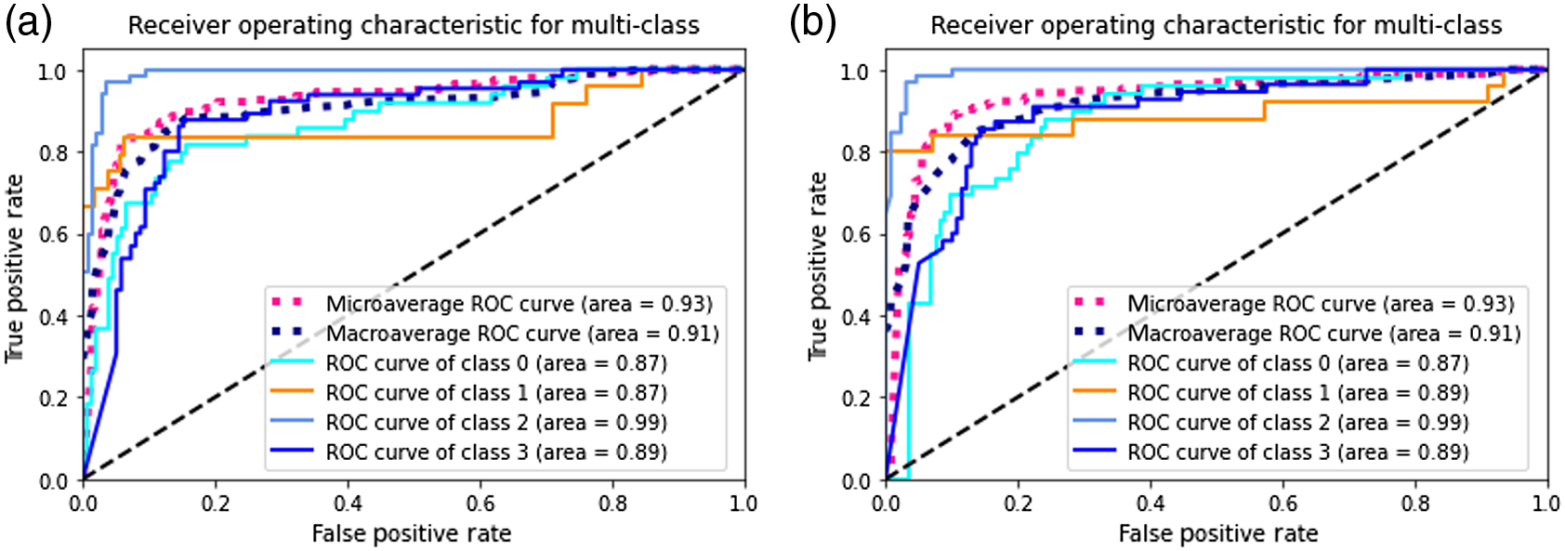
ROC curve of each class and micro-/macroaverage combined: (a) trained with RUS and data augmented balanced dataset using focal loss and (b) trained with bootstrap aggregation ensemble method (In this figure, classes 0 to 4 represent class benign, malignant, normal, and premalignant, respectively).

In order to further improve the performance of CNN classifier, we applied focal loss to the RUS and data augmented dataset. The focusing parameter and weighting factor of focal loss was 2 and 0.25, respectively. The confusion matrix results are shown in Fig. 4(a) and the ROC curve is shown in Fig. 5(a). By combining both data-level and algorithm-level approaches, better performance achieved on minority classes.

We also tried the bootstrap aggregation ensemble method. The normal and premalignancy classes of the dataset were first undersampled to 600, and randomly separated to 5 subsets; the benign class was randomly separated to 2 subsets. Then each subset of the majority classes, combining with one of the benign subset and minority malignancy create one balanced subdataset and repeated to generate five balanced subdatasets. The balanced subdatasets were used to train multiple classifiers. We used VGG19 as the base network to train, using the same settings (e.g., learning rate, epoch, and batch size) as before. Each classifier was trained with focal loss. The final decision was the combination of the multiple classifiers. The result of bootstrap aggregation ensemble method is shown in Fig. 4(b) (confusion matrix) and Fig. 5(b) (ROC curve of each class and micro-/macroaverage combined). This method helps reduce variance and avoid overfitting with the idea that multiple learners usually outperform a single learner, but the disadvantage is computationally expensive.

## 4 Discussion

It is common for oral cancer image datasets collected from high-risk populations to have an uneven number of examples among different classes. This data imbalance usually complicates the learning process, especially for the minority classes, and results in bad performance.

In this study, different approaches, including weight balancing, data augmentation, undersampling, focal loss, and bootstrap aggregation ensemble methods, have been investigated to handle the imbalanced, multi-class learning problem. We used weight balancing and focal loss on the original unbalanced dataset for comparison, then balanced the dataset using data augmentation and RUS, and integrated algorithm-level methods to the generated balanced dataset. The ensemble method bootstrap aggregation has also been investigated and shown to further reduce class bias at the cost of longer required training times. The experimental results show that by applying both data-level and algorithm-level approaches to the deep learning training process, good performance can be achieved on imbalanced multi-class oral cancer image datasets. Although the total accuracy has not changed much, the performance of the minority classes, which were difficult to distinguish at the beginning, has been greatly improved. The accuracy of premalignancy class is also increased, which is ideal for screening applications. Although we used VGG19 net in all the experiments for a fair and meaningful comparison, other convolutional neural networks should also be able to apply these mentioned approaches to handle the unbalance problem for oral cancer datasets.

This study may provide an important basis for helping understand the influence of unbalanced dataset on oral cancer deep learning classifier and application of the classifier in the screening of oral cancer among high-risk population.
